# A comprehensive quantitative lifecycle cost and environmental impact analysis model for computing infrastructure

**DOI:** 10.1016/j.mex.2024.103009

**Published:** 2024-10-24

**Authors:** Kezhuo Ma, Yu Zhou

**Affiliations:** a Peking University HSBC Business School, University Town Peking Campus Lishui Rd, Shenzhen, 518000, PR China; b Shenzhen Greater Bay Financial Institute, Baisong Rd, Longhua District, Shenzhen, 518000, PR China; c Faculty of Engineering and Information Technology, the University of Melbourne, VIC 3010, Australia

**Keywords:** Computing infrastructure, Lifecycle cost analysis, Greenhouse gas emission, Emission factor method, A quantitative model for cost and environmental impact of computing infrastructure

## Abstract

This paper presents a comprehensive quantitative model for quantitative assessment of the lifecycle costs and environmental impacts of computing infrastructure, with a focus on internet data centers (IDCs) and high-performance computing (HPC) facilities. The key innovation lies in the integration of interdisciplinary cost evaluation and carbon emission methods for the establishment of this quantitative model. This framework, which outlines key cost components and carbon emission factors, enables the calculation of total costs, electricity expenses, and greenhouse gas emissions throughout the lifecycle of infrastructure. With IDCs as a case study, the research clarifies the intricate cost structure associated with equipment procurement, energy usage, land acquisition, and operational expenses. This paper provides an in-depth understanding of the cost structure and environmental impact of computing infrastructure in support of sustainable decision-making in its development.

• Based on established cost estimation methods, such as Lifecycle Cost Analysis (LCCA) and the Analogous Estimating Method, this study examines costs across construction and operation phases.
• The Emission Factor Method is used to quantify environmental impact, emphasizing the significance of regional energy mix and power usage effectiveness (PUE).

## Graphical abstract

**Figure:**
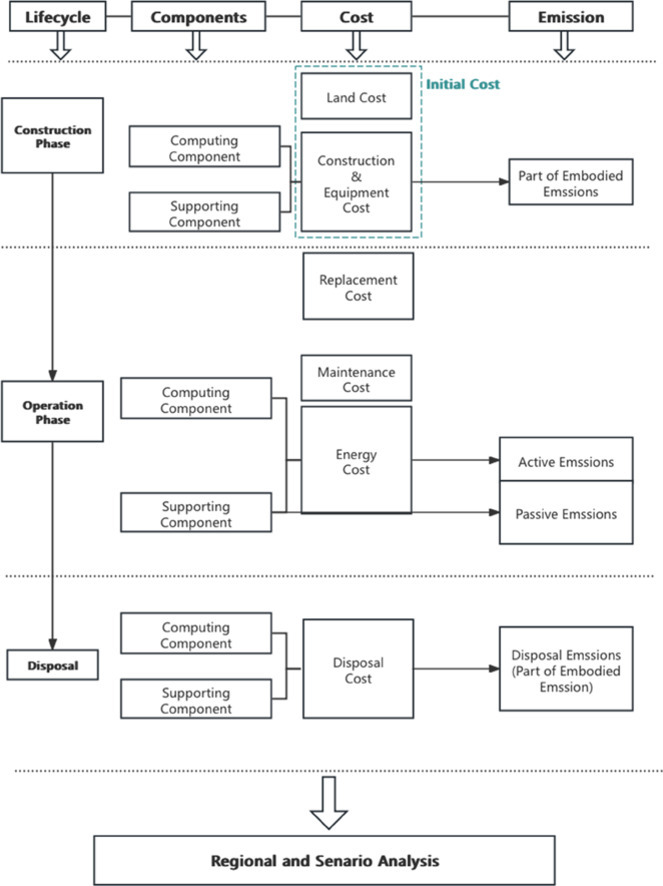

Specifications table

**Table:** 

Subject area:	Environmental Science
More specific subject area:	Environmental Impact Assessment
Name of your method:	A quantitative model for cost and environmental impact of computing infrastructure.
Name and reference of original method:	The cost evaluation is based on Lifecycle Cost Analysis (LCCA), while the missing details would be estimated through the Analogous estimating method.
	In terms of environmental impact assessment, Life Cycle Assessment (LCA) method is used to quantify the total emission of study subject.
Resource availability:	The data supporting the findings of this study are available within the article.

## Background

The recent advancements in Artificial Intelligence (AI), especially Generative Artificial Intelligence (Gen AI) are reshaping industries through techniques such as machine learning, natural language processing, computer vision, and robotics [1]. These technologies contribute to improvements in several aspects: they optimize manufacturing, enhance healthcare with personalized medicine and precise diagnostics [2], and improve financial services through automated decision-making and fraud detection [3]. In scientific research, Gen AI expedite discoveries through the analysis of complex datasets and promotion of interdisciplinary collaborations [4], driving efficiency, productivity, and scientific progress [5]. Consequently, AI is shaping a future of profound societal transformation.

The sustainable development and application of Gen AI necessitate robust computing power and Information and Communications Technology (ICT) infrastructure, including Internet Data Center (IDC), data communication networks, and terminal devices [6]. As Gen AI becoming increasingly integral to modern society, the demand for computational resources escalates, with IDC as the backbone of ICT infrastructures, providing the necessary computing power and storage capacity [7]. However, the rising demand for computing power demands presents significant challenges in terms of energy usage and environmental impact [[8], [9]].

In recognizing of the importance of digital infrastructure development, many countries worldwide have published corresponding strategies. The United States has outlined its strategy in the "National Artificial Intelligence Research and Development Strategic Plan” [10], while France has introduced the National Strategy for AI as a part of ”France 2030” plan [11]. Meanwhile, leading ICT companies, such as Amazon Web Services (AWS), Google Cloud Platform, Microsoft Azure, and Huawei Cloud, are expanding their computing infrastructure, with the IDC and its services as their primary products and services. These national strategies, coupled with the proactive effort of ICT companies to react to market demand, will bring an increase in IDC construction projects. The construction and operation will generate a great amount of energy usage and costs. The growth in energy usage due to IDC expansion cannot be offset by energy efficiency improvements, which will exacerbate the challenge of cost management, energy usage and environmental impact. In response, a comprehensive understanding of the costs and environmental impacts associated with computing infrastructure is essential. The quantification of these factors enables stakeholders to make informed decisions, with the objective of promoting green computing power and sustainable development of computing infrastructure.

This study aims to propose a quantitative approach to evaluate the cost and environmental impact of computing infrastructure, with IDC as a case of study. Through these efforts, the aim is to pave the way for a more sustainable digital future. This paper is organized as follows: Background section provides context for the study and highlights the importance of understanding of the costs and environmental impacts associated with computing infrastructure. Method details section is divided into four key parts: first, the Cost Evaluation framework is outlined, followed by the application of a Life Cycle Assessment (LCA) for environmental impact. Third, the development of a conceptual model is introduced, providing a theoretical foundation. Fourth, the paper details the construction of a comprehensive quantitative model, including the quantitative formulas used for calculating both costs and environmental impact. In the Method Verification section, the model is applied to three scenarios, and the evaluated results from model would be conducted cost and emission distribution analysis. Finally, the paper offers suggestions for the practical application of this method and proposes directions for future studies.

## Method details

This paper presents a quantitative method and conceptual model for the assessment of the construction costs and environmental impact of IDC, a type of computing infrastructure. IDCs are complex, multi-layered systems primarily encompassing components such as equipment, energy, land, operation and maintenance costs (including personnel expenses), among others [12]. The quantification of the cost of IDCs requires a comprehensive evaluation of these various components. In terms of equipment cost, a significant expenditure is the procurement of computing equipment and other support systems [13]. The electricity consumption of computing equipment directly affects the provision of computational resources, while electricity costs for support systems include power required to maintain environmental conditions for proper equipment operation [14]. Thus, energy usage includes both the electricity supply for computing equipment and for the support systems, which mainly involve equipment such as air conditioning and refrigeration systems. Land costs depend on the geographical location of the IDC, while operational expenses encompass personnel training, equipment maintenance, and other aspects. Additionally, the electricity generated by different resources (such as fossil fuels, wind, solar or other renewable energy) significantly impacts carbon emissions and, consequently, environmental sustainability [15].

1. Cost Evaluation

This paper aims to evaluate the cost of IDC. As a first step, we review various cost evaluation and management methods widely used in engineering construction projects. The following paragraphs review methods involved in the establishment of the conceptual model for the assessment of the cost of IDCs.

• LCCA [16] is used to evaluate costs through the entire project lifecycle, including conception, design, construction, operation, and eventual decommissioning, instead of the traditional cost estimation method. LCCA considers both direct and indirect project-related costs, as well as maintenance and disposal costs, with the objective of covering costs through the whole lifecycle [17]. This method is widely used to select the best alternative, considering financial and environmental aspects.
• A key concept in LCCA is the life boundary [18]. Different life boundary settings can lead to varying results of cost and environmental impact evaluation. Setting the life boundaries before evaluation ensures all relevant costs and benefits are considered, with those outside the defined boundaries excluded. In the analysis of the whole lifecycle of a project, the boundaries typically include the entire lifecycle of an asset. Meanwhile, for some narrow-scale studies, LCCA can also be used to evaluate project costs between set life boundaries, such as sole focus on the construction and operation phases.
• Analogous Estimating Method [19] is useful for the production of cost estimates without enough available information. This method utilizes historical data from previous projects to estimate costs for the current project or parts of it. This method is normally used during the early planning phase with limited detailed information. The accuracy of this method depends on the similarity between the previous project and the estimated project and the availability of detailed data from past projects.

This paper aims to establish a conceptual model for computing infrastructure to quantify the cost and environmental impact in set life boundaries, mainly based on LCCA. The cost evaluation of IDCs in this study will focus on the construction, operation, and disposal phases of the whole project lifecycle. For cost components where precise calculation parameters are difficult to obtain, the Analogous Estimating Method will be used. The construction phase costs include land acquisition costs, computing components and support components (with building construction costs currently excluded). In the operation phase, the evaluation will consider energy costs and maintenance costs (including personnel costs). Additionally, the disposal cost of computing infrastructure components will also be taken into consideration.

2. Carbon Emission

• Life Cycle Assessment (LCA)

In this study, a comprehensive Life Cycle Assessment (LCA) is employed to evaluate the environmental impacts associated with the entire lifecycle of the building materials and processes. The LCA methodology adheres to ISO 14040:2006 standards [20], encompassing four key stages: goal and scope definition, inventory analysis, impact assessment, and interpretation. The primary objective of the LCA is to quantify environmental impacts, with a particular focus on global warming potential, resource depletion, and energy usage. The scope includes all stages, from raw material extraction, transportation, and construction to operation and end-of-life disposal. Inventory analysis involves the systematic collection of data to accurately represent the inputs (e.g., materials, energy) and outputs (e.g., emissions, waste) across the building's lifecycle. The impact assessment phase translates this data into specific environmental impact categories, with an emphasis on embodied carbon and operational energy use. The interpretation of the results provides critical insights into the building's environmental performance, highlighting key areas for improvement and offering recommendations to minimize the overall environmental footprint. This LCA approach ensures a holistic understanding of the building's sustainability profile in support of informed decision-making throughout the design and construction processes.

3. The Conceptual Model of Lifecycle Cost and Environmental Impact Analysis Model for Computing Infrastructure

**Fig. 1:**
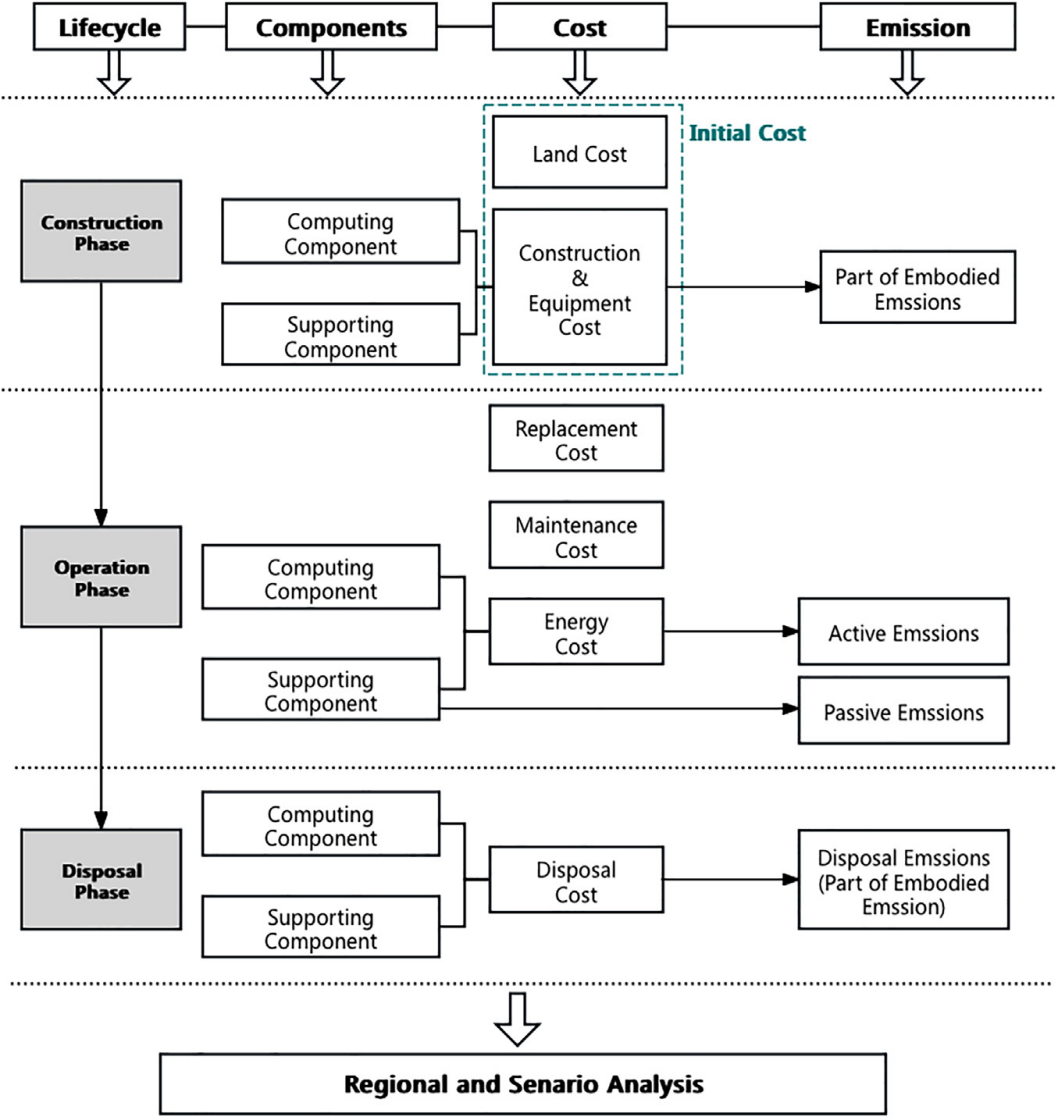
The Conceptual Model of Comprehensive Lifecycle Cost and Environmental Impact Analysis Model for Computing Infrastructure.

Based on the quantification methods outlined above, and sensible choice for lifecycle boundaries, this paper establishes a conceptual model for quantifying the cost and environmental impacts of IDC, shown in Fig. 1. The considered lifecycle includes construction, operation and disposal phases.

## The comprehensive quantitative model

This paper aims to establish a quantitative model of the evaluation of the environmental impact and cost of computing infrastructure for a defined period. The model can be applied to assess the whole life cycle under ideal conditions, such as gaining access to complete supply chain data and life cycle monitoring or forecast data.

### Lifecycle analysis for GHG emissions

**Fig. 2:**
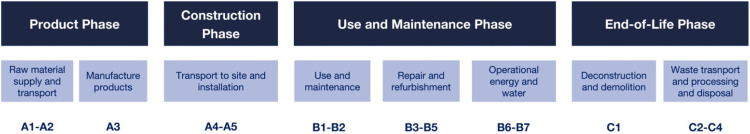
Life cycle assessment phase.

In terms of environmental impact, this paper applies the Life Cycle Assessment (LCA) methodology, which is a standardized methodology used to quantify the environmental impacts associated with all stages of a product lifecycle, from raw material extraction (cradle) to disposal or recycling (grave), as following Fig. 2.

Fig. 2 illustrates the phases of a product's lifecycle, segmented into four main phases: Product Phase, Construction Phase, Use and Maintenance Phase, and End-of-Life Phase. Each phase is further divided into specific stages, denoted by standardized lifecycle stage codes (A1–C4), which are essential for environmental impact assessments and life cycle analysis (LCA).

• Product Phase (A1-A3): This phase includes the extraction and transportation of raw materials (A1-A2) and the manufacturing of products (A3).
• Construction Phase (A4-A5): This phase includes the transport of products to the construction site (A4) and the installation process (A5).
• Use and Maintenance Phase (B1-B7): This phase involves the operational phase of the product, including energy and water use (B1-B2), maintenance and repairs (B3-B5), and operational energy and water consumption (B6-B7).
• End-of-Life Phase (C1-C4): This phase focuses on deconstruction (C1), and the transport, processing, and disposal of waste (C2-C4).

This framework provides a comprehensive approach to the assessment of the environmental impacts at each stage of a product's lifecycle. It is crucial for sustainability evaluation and informed decision-making in construction and product development projects. The focus of this paper covers the stages from A4 to C4, as seen in Fig. 2.

The focused period in this paper covers through A4 to C4 in Fig. 2.

#### Emission components

To conduct the environmental impact quantification, carbon emission is classified as active emission, embodied emission and passive emission.

1). Active emission

Active emissions represent the carbon emissions associated with energy usage during the operational phase, which are influenced by specific time periods. The carbon emissions related to mixed energy sources and supporting facilities (e.g., cooling, lighting) are variable and depend on the defined evaluation period. For instance, changes in the regional energy mix ratio due to seasonal shifts or fluctuations in the regional energy market can lead to variations in carbon intensity factors. Additionally, seasonal variations in temperature and sunlight would affect the energy consumption of supporting systems. Furthermore, energy usage for computing infrastructure fluctuates with system load and usage, contributing to the overall variability in emissions.

2). Embodied emission

The embodied carbon of item is generally considered a fixed cost, assuming no additional infrastructure or hardware is introduced during the considered period. This embodied carbon encompasses the carbon emissions generated during the manufacturing, installation, and disposal phases of the equipment. To assess the embodied carbon over the item lifespan, it can be amortized, yielding the embodied carbon per unit of time throughout its whole lifespan. When calculating the embodied carbon, it is quantified based on the projected time frame and embodied carbon per unit of time. The quantification of embodied carbon encompasses both the computing infrastructure and the support systems (buildings, cooling and power and other associated fixed infrastructure).

3). Passive emission

Passive emissions are the indirect greenhouse gas emissions that occur due to the inherent characteristics of the building, such as its design, materials, and orientation, without the active involvement of energy-consuming systems. These emissions result from natural processes like heat loss through poorly insulated walls or heat gain from solar radiation through windows. Passive emissions are influenced by factors such as the building's thermal mass, insulation, and ventilation design, which details could be found in Appendix Table 1.

**Table 1:** The evaluated results of life cycle cost for 3 scenarios by model.

	Scenarios 1 Shenzhen, China	Scenarios 2 Guizhou, China	Scenarios 3 North Virginia, USA	Unit
**Construction phase**	51.44	51.44	51.34	million CNY/year
Land cost	0.13	0.03	0.12	
Computing component	20.52	20.52	20.52	
Support component	30.79	30.79	30.79	
**Operation Phase**	13.08	9.95	42.83	million CNY/year
Energy cost	7.45	5.67	29.98	
maintenance cost	6.15	4.68	12.85	
**Disposal**	7.17	6.81	10.47	million CNY/year
***Life cycle cost***	71.68	68.20	104.65	million CNY/year

#### Calculating formulas

According to the above section, the carbon model for environmental impact includes three forms:

(1) $$E_{t}^{p}=\;E_{a}^{p}+E_{e}^{p}+E_{p}^{p}\;$$

where $E_{t}^{p}$ is the total carbon emission for the period $p,\;E_{a}^{p}$ is the active emission for the same period covering all items, $E_{e}^{p}$ is the embodied emission for that period, and $E_{p}^{p}$ is the passive emission through the same period.

1). Active emission

The active emission illustrates the sum of all carbon emission conducted by energy usage by the active infrastructures, including computing infrastructure and supporting infrastructure during period p.

(2) $$E_{a}^{p}=\sum_{1}^{items}\sum_{1}^{p}E_{a_{computing}}+\;\sum_{1}^{items}\sum_{1}^{p}E_{a_{support}}\;$$

For individual items, the $W_{computing}^{p}$ presents the sum of energy usage of computing equipment, and the $W_{support}^{p}$ presents the sum of energy usage of all facilities to support and host the computing equipment operating steady.

The carbon emission for energy usage of each item could be calculated by energy usage and carbon intensity factors.

(3) $$E_{a}x^{p}=\;W_{x}^{p}\times C_{a}^{p}\;$$

where $E_{a}x^{p}$ presents the active emission of energy usage of item x during period p. $W_{x}^{p}$ is the energy usage of item x during period p, and $C_{a}^{p}$ is the carbon intensity factors (various by different energy resource structure) for the electricity usage.

2). Embodied emission

The embodied emission could be presented in the same method as active emission:

(4) $$E_{e}^{p}=\sum_{1}^{items}\sum_{1}^{p}E_{e_{computing}}+\;\sum_{1}^{items}\sum_{1}^{p}E_{e_{support}}\;$$

$E_{e}x$ presents embodied emission of item x, which is the sum of carbon released during the manufacturing, installation, and disposal phases, and the T is the lifespan of item x.

Aim to calculate the embodied emission for considered period, the embodied emission would be amortized over the lifespan as below. $E_{e}x_{unit}\;$presents the embodied carbon in unit time of item x*.*

(5) $$E_{e}x_{unit}=\frac{E_{e}x}{T}\;$$

3). Passive emission

Estimating passive emissions need to calculate the heat loss and gain through the building envelope, which lead to the energy demand and the associated emissions.

i). Heat Loss $Q_{loss}$
  (6) $$Q_{loss}=U\times A\times \Delta T\times t$$

• $Q_{\text{loss}}$: Heat loss through the building envelope (in joules or BTU).
• U: U-value of the building component (W/m²·K), representing the thermal transmittance.
• A: Surface area of the building component (m²).
• ΔT: Temperature difference between the inside and outside (°C or K).
• t: Time period over which the heat loss is calculated (hours).

ii). Solar Heat Gain
  (7) $$Q_{gain}=A_{window}\times SHGC\times I\times t$$

• $Q_{gain}$: Solar heat gain through windows (in joules or BTU).
• $A_{window}$: Area of windows (m²).
• SHGC: Solar Heat Gain Coefficient of the windows (dimensionless).
• I: Incident solar radiation on the window (W/m²).
• t: Time period over which the heat gain is calculated (hours).

iii). Total Passive Emissions

Passive emissions typically result from the natural processes of heat transfer, solar gain, and other environmental interactions. The total passive emissions can be calculated by translating the total heat gain and loss into the energy required for heating or cooling, then multiplying by the carbon intensity of the energy source used for conditioning the space.

(8) $$E_{p}=\left(\frac{Q_{loss}-Q_{gain}}{\eta }\right)\times \;CI\;$$

• $E_{p}$: Passive emissions (kg CO₂eq).
• η: Efficiency of the heating/cooling system.
• CI: Carbon intensity of the energy source (kg CO₂eq/kWh).

### Lifecycle cost analysis

In assessing the lifecycle cost of the whole computing infrastructure, this model considers the construction, operation, and disposal phases. The computing infrastructure is categorized into computing components and support components. The computing components include all items responsible for generating computing capabilities. In contrast, the support components encompass all facilities essential for maintaining the stable operation of the computing components, such as cooling systems, power supply, network infrastructure, heating, lighting, and other building systems.

#### Phases of analysis

1). Construction phase

During the construction phase, the model accounts for the costs associated with land acquisition, construction, and equipment for both the computing and supporting components. These expenses constitute the initial cost of the entire computing infrastructure. With the aim to simplify the calculation, the initial cost will be spread evenly to the unit time.

2). Operation phase

During the operation phase, the model accounts for the energy consumption and maintenance costs associated with the computing infrastructure. Additionally, replacement costs are incurred when components exceed their operational lifespan.

3). Disposal

Disposal cost refers to the expenses incurred at the end of an asset useful life, including decommissioning, removal, waste management, recycling, and site restoration. These costs are essential to be included in the lifecycle cost analysis to ensure a comprehensive evaluation of long-term financial implications.

#### Calculating formulas

According to the above section, the lifecycle cost analysis of Computing Infrastructure contains three forms;

(9) $$C_{t}^{p}=C_{c}^{p}+C_{o}^{p}+C_{d}^{p}\;$$

where $C_{t}^{p}$ is the lifecycle cost of computing infrastructure for the time period $p,\;C_{c}^{p}$ is the cost of computing infrastructure during construction phase for the same period, $C_{o}^{p}$ is the cost of computing infrastructure during operation phase for that period, and $C_{d}^{p}$ is the disposal cost of computing infrastructure through time period p.

1). Cost of construction phase
  (10) $$C_{c}^{p}=C_{land}^{p}+C_{computing}^{p}+C_{support}^{p}\;$$

where $C_{c}^{p}$ presents the total cost of construction phase amortized in period $p,\;C_{land}^{p}$ presents the cost of land acquisition in period $p,\;C_{computing}^{p}$presents the cost of computing components in period $p,\;C_{support}^{p}$presents the cost of support components in period p.

i). Land cost

Land cost $C_{land}$ could be gained by below formula:

(11) $$C_{land}=A\times P_{industrial}\;$$

whereA is the land use area of Internet Data Centre, and $P_{industrial}$ is the unit price of industrial land use depending on the local land market (where the land use of Internet Data Centre belongs to industrial).

Land acquisition costs are a one-time expenditure. To simplify the calculation, these costs will be amortized based on the duration of land use $T_{land\;use}$. So that $C_{land}^{p}$, the land cost during the evaluated period p, could be gained by

(12) $$C_{land}^{p}=\frac{C_{land}}{T_{land\;use}}\times p\;$$

ii). Cost of components

The components of computing infrastructure could be classified as computing components and support components. The total cost of all computing infrastructure components during defined period pcould be gained as flow:

(13) $$C_{c}^{p}=\sum_{1}^{items}C_{computing}^{p}+\sum_{1}^{items}C_{support}^{p}\;$$

The cost of the construction components can be amortized into the cost per unit time according to the lifespan of component to simplify the calculation.

(14) $$C_{x}^{p}=\frac{C_{x}}{T}\times p\;$$

where $C_{x}^{p}$ presents the cost of item xof construction phase amortized in period p,Tis the lifespan of item x.

According to the Indian data centre consult [21], the computing components occupies approximate 40% total component cost. So that the total component cost of computing infrastructure could be calculated by

(15) $$C_{c}^{p}=\frac{C_{computing}^{p}}{0.4}\;$$

2). Cost of operation phase

The operational phase costs include replacement costs, energy consumption costs, and maintenance costs.

(16) $$C_{o}^{p}=C_{replacement}^{p}+C_{energy}^{p}+C_{maintenance}^{p}\;$$

Where $C_{o}^{p}$ presents the total cost of operation phase in period $p,\;C_{energy}^{p}$ presents the cost of energy usage in period $p,\;C_{maintenance}^{p}$presents the cost of maintenance in period p.

Since the allocation of costs for the components has been accounted for in the previous section, the replacement of components has already been included in that calculations. Therefore, the following calculation will not duplicate component replacement cost again. So that in the later calculation, the formula for operational phase costs could be presented as

(17) $$C_{o}^{p}=C_{energy}^{p}+C_{maintenance}^{p}\;$$

i). energy usage cost

The cost of energy usage is obtained from the total energy consumption and the unit price of electricity from the regional grid.

(18) $$C_{energy}^{p}=W_{t}^{p}\times P_{regional}\;$$

where $C_{energy}^{p}$ is the cost of energy usage during defined period p, the $W_{t}^{p}$ is the total energy consumption of the computing infrastructure for the same period p, and the $P_{regional\;grid}$ is the price of regional grid electricity.

The total energy consumption of the computing infrastructure comprises the energy usage of both the computing components and the support systems.

(19) $$W_{t}^{p}=W_{computing}^{p}+\;W_{support}^{p}\;$$

where $W_{computing}^{p}$ is the energy consumption of the computing components for the period p, and $W_{support}^{p}$ is the energy consumption of the support components for the period p.

Moreover, the energy usage calculations for the support component already incorporate the building's passive energy consumption, which refers to heat loss or gain due to the inherent characteristics of building materials, design, and prevailing climatic conditions. Consequently, there is no need for a separate calculation of these passive effects. However, if a separate calculation is desired, the formulas outlined in Section 4.1.2 provide the necessary guidance.

The Power Usage Effectiveness (PUE) is the ratio of all energy consumed by the whole computing infrastructure to the energy consumed by the computing component.

(20) $$PUE^{p}\;=\;\frac{W_{t}^{p}}{W_{computing}^{p}}\;$$

(21) $$W_{computing}^{p}=\sum_{1}^{item}\eta^{p}P_{computing}^{p}\times p\;$$

Where $\eta^{p}$ is the average load of computing components during the considered period p, and the $P_{computing}^{p}$ is the average power of computing components during the same period.

Combined the below formulas, the total energy usage of the whole computing infrastructure during period p is expressed as

(22) $$W_{t}^{p}=\sum_{1}^{item}\eta^{p}P_{computing}^{p}\times p\times PUE^{p}\;$$

ii). Maintenance

As mentioned in the above section, the replacement cost is already included in the amortization calculation for components, so that the cost of operation phase during period p could be calculated as

(23) $$C_{o}^{p}=C_{energy}^{p}+C_{maintenance}^{p}\;$$

Based on the labor markets and energy prices in different regions, the ratio of energy costs to operational phase costs $r_{energy}^{p}$ will vary.

(24) $$r_{energy}^{p}=\frac{C_{energy}^{p}}{C_{o}^{p}}\;$$

3). Disposal cost

According to a case study of an industrial building [22,23], disposal costs account for approximately 10.2% of the total life cycle cost, which includes land acquisition, operation and maintenance, energy consumption, construction, and disposal costs. Thus, for the calculation of model, we could have a reasonable estimation is that the disposal costs represent 10% of the total life cycle cost.

## Quantitative result analysis

This paper utilizes public data regional data from China and USA to further verify and showcase the feasibility of this model. In the following Method Verification section, it would conduct evaluation results for three scenarios (two for China, one for USA) from the established quantitative model, and present the preliminary regional analysis from lifecycle cost and environmental impact aspects. However, detailed regional and scenario analysis will be in the scope of future studies as this work is a method study.

While this study primarily discusses the above comprehensive quantitative model, certain data will be needed to conduct quantitative analysis. Appendix Table 2 suggests a List of data for computing infrastructure cost and environmental impact quantification.

**Table 2:** The emission of 2 IDC scenarios in Shenzhen and Guizhou.

	Scenario 1 Shenzhen, China	Scenario 2 Guizhou, China	Scenario 3 North Virginia, USA	Unit
Total energy consumption	62.782	60.000	47.682	TWh/year
Emission factor	0.445	0.420	0.283	kgCO_2_/kWh
**Active Emission**	27.938	25.200	13.494	million kgCO_2_/year
**Embodied emission**	3.339	3.339	3.339	million kgCO_2_/year
***Total emission***	31.277	28.539	16.833	million kgCO_2_/year

## Method verification

For the quantitative calculation in this study, the presumed scale of the IDC was set at 1000 server racks (based on the upper limit for newly constructed data centers in Guangdong Province government document), with power supplied directly from the local regional grid. Drawing on the actual case of the China Mobile Sanming data center (1000 server racks, 5.35 acres, approximately 3566m^2^) [24], a comparison method was employed to establish a presumptive land area of 3600m^2^ for the 1000-server-rack configuration. This served as the basis for quantifying the total cost and environmental impact of the IDC. It is assumed that the IDC is equipped with uniform model of servers, specificallyell PowerEdge R710 2U rack server. This simplifies the calculations, as we can estimate the total energy use and costs of the IDC by using key parameters including the server's model, power consumption, price, lifespan, and carbon footprint in our quantitative model. The server rack is 42U and a single server rack could accommodate 21 servers with a 2U specification. Thereby, the computing components of IDC would have 21000 rack servers.

This paper will evaluate the costs and emissions of three IDC scenarios located in different regions with varying land resources, industrial land prices, energy mixes, and labor markets. These differences lead to variations in energy prices, emission factors, and the ratio between energy costs and maintenance costs, which will be considered in the following calculations. The basic information of the three scenarios are outlined below.

Scenario 1: Shenzhen, China

Shenzhen, a major technology hub in southern China, is characterized by high labor costs and limited land availability. Industrial land prices are among the highest in the country, and the energy mix is primarily reliant on fossil fuels, though renewable energy sources are growing. These factors result in higher operational costs for data centers, particularly due to the region's dense urban development and high energy prices.

Scenario 2: Guizhou, China

Guizhou, located in southwestern China, offers a contrasting environment with lower labor costs and abundant land resources. Industrial land prices are significantly lower than in Shenzhen, making it an attractive region for large-scale data center development. The energy mix in Guizhou is dominated by hydropower, which reduces both energy costs and emissions, making it a more environmentally sustainable option. The energy structure in Guizhou is characterized by a higher proportion of clean energy, with 52% of its energy derived from non-fossil sources.

Scenario 3: North Virginia, USA

North Virginia is a well-established data center hub in the United States, with a highly skilled labor market and strong technological infrastructure. Labor costs are higher than in China, but the region benefits from a relatively stable energy mix, consisting of natural gas, nuclear power, and an increasing share of renewable energy. While industrial land prices are higher in proximity to major metropolitan areas, North Virginia's strategic location near key internet exchange points offers significant operational advantages for data centers.

1). Cost
i). Key details for the cost of construction phase

There are significant differences in land resource costs and energy conditions between scenarios. The industrial land price in Guizhou is 390 CNY/m² [23], 1753 CNY/m² in Shenzhen [24], and $236.33 per m^2^ (1683 CNY/m²) in North Virginia [25].

According to Article 12 of the Provisional Regulations of the People's Republic of China on the Assignment and Transfer of the Right to the Use of State-Owned Land in Urban Areas [26], the land use term for industrial land is uniformly set at 50 years across the country. To simplify the calculations, the industrial land use duration in three scenarios are all assumed to be 50 years. In contrast, the lifespan of computing components is 4 years.

When calculating the costs of land and components, the fixed costs are amortized to determine the cost per unit of time (annual average), which includes the component replacement cost. Given that computing components account for 40% (in section 4) of the total component cost, which allows to estimate of the total cost of the all the IDC components.

ii). Key details for the cost of operation phase

According to “China 5G and Data Center Carbon Emissions Outlook 2035” [27], the Power Usage Efficiency (PUE) in Shenzhen and Guizhou is 1.58 and 1.51, respectively. For the USA scenario, the new data center energy efficiency bill in Virginia House in 2024 [28], the PUE of facilities should be reached 1.2.

By incorporating the server rack parameters mentioned earlier, the total energy consumption of the IDCs in Shenzhen, Guizhou and North Virginia can be calculated as 62.78 TWh/year, 60.00 TWh/year, and 47.68 TWh/year respectively.

The energy usage cost would be evaluated based on Notice of the National Development and Reform Commission on Provincial Power Grid Transmission and Distribution Prices and Related Matters in the Third Regulatory Cycle [29] in May 2023, and the regional grid price are separately sorted in Appendix Tables 3 and 4. Appendix Table 3 details the regional electrical grid price of Chine (excluding Shenzhen) categorized by province (scenario 2). Additionally, Appendix Table 4 supplies the electrical grid price of Shenzhen city (scenario 1). The industrial electricity price of Virginia could be found in Electricity Data Brower from U.S. Energy Information Administration [30], which shows that the industrial electricity price of Virginia in 2023 is $8.83 cents/kWh (scenario 3).

According to the analysis of data centre operating costs in the 2021 China Data Centre Report released by Savills [31], the energy cost accounts for the main part of operating expenses in China(scenario1&2), accounting for 57%, and the rest of the expenses (maintenance cost) incurred during operation account for 43%. Based on the USA government labour report [32], we assume the ratio of energy costs to operational phase costs is 70% in Virginia (scenario 3).

Furthermore, based on the proportion of energy costs in the Operation Phase as detailed in Section 4 and the previously calculated energy costs, the total cost of the Operation Phase can be determined.

iii). Key details of disposal cost

Finally, according to Section 4, where the cost during the disposal phase accounts for 10% of the total lifecycle cost (considering construction, operation, and disposal phases), we can derive the overall lifecycle cost.

iv). Evaluation results of the life cycle costs for three scenarios

Table 1 summarizes the calculated costs for each phase of the lifecycle covered by the model on an annual basis (million CNY/year).

According to the results presented in Table 1, the land cost for Scenario 1 and Scenario 3 are close, the industrial land prices in Shenzhen and North Virginia are both far high away than Guizhou (Scenario 2). The cost of land for Scenario 1 Shenzhen is 449% of that for Scenario 2 Guizhou, while the energy cost for Scenario 1 is 131% of the corresponding cost for Scenario 2 Although the IDC in North Virginia (Scenario 3) have the lowest PUE and total energy consumption, the electricity costs are approximately three to four times higher than those in Scenario 1 and Scenario 2 due to significantly higher unit electricity prices.

In Scenario 3 (North Virginia), the total lifecycle cost is the highest, primarily due to the significantly higher operational costs compared to the other two scenarios. The energy costs in the operational phase of Scenario 3 are substantially higher than those in the Shenzhen and Guizhou scenarios, accounting for 28.63% of the total lifecycle cost. In contrast, energy costs in the other two scenarios make up less than 10% of the total cost.

When considering the total lifecycle costs, the data center IIDC) in Shenzhen incurs an additional expense of 3600 thousand CNY per year compared to the IDC in Guizhou. Both scenarios 1 and 2 are situated in China, and even though the total lifecycle costs differ due to energy prices, the labor market, and energy efficiency, their total costs are still significantly lower than those of scenario 3, located on another continent in USA.

**Fig. 3:**
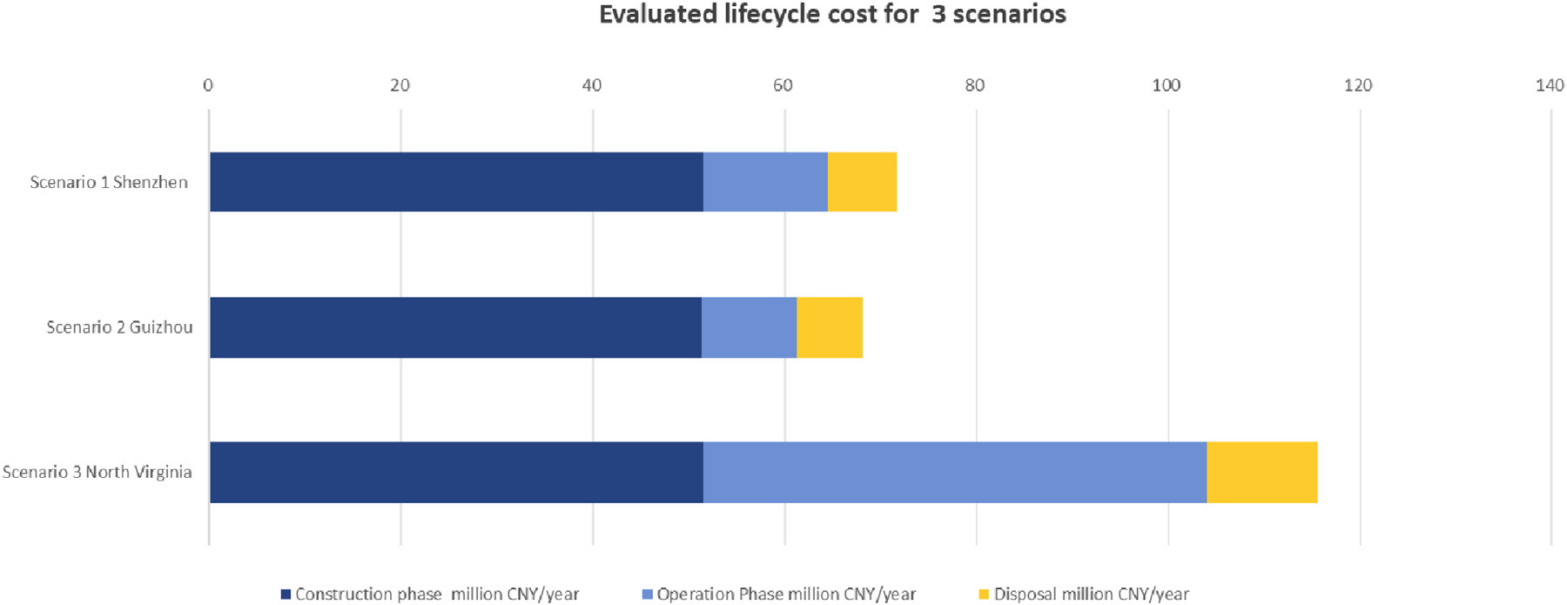
Evaluated lifecycle cost for 3 scenarios (million CNY per year).

**Fig. 4:**
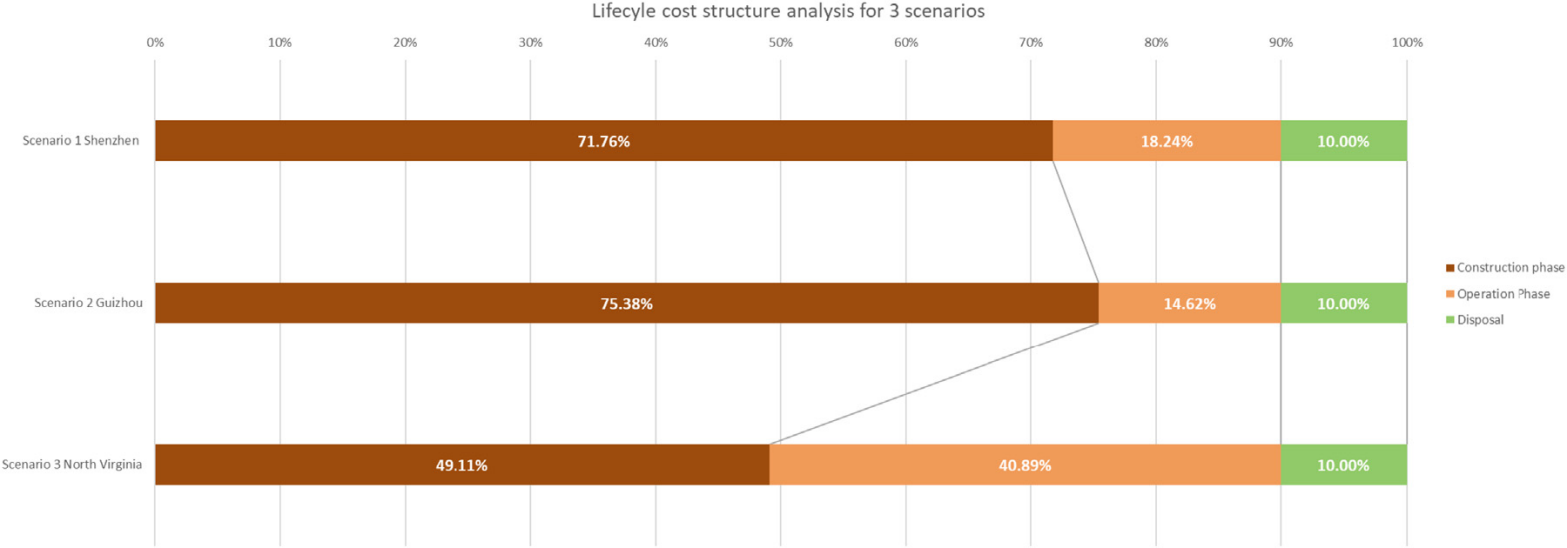
Lifecycle cost structure for 3 scenarios.

The evaluation results of the total lifecycle cost (Fig. 3) and the cost distribution (Fig. 4) for the three scenarios are analyzed in the following paragraphs.

The analysis of evaluated lifecycle costs (Fig. 3) and cost structure (Fig. 4) across the three scenarios (Shenzhen, Guizhou, and North Virginia) provides critical insights into the regional distribution of expenses across the construction, operation, and disposal phases, with the assumption that the disposal cost occupies a fixed 10% of the total lifecycle cost in each scenario.

In Scenario 1 Shenzhen, the construction phase constitutes the majority of the total lifecycle cost, accounting for 71.76%, with an evaluated cost of 51.44 million CNY/year. The operation phase follows with 18.24%, corresponding to 13.08 million CNY/year. Given the assumption that disposal costs account for 10%, the disposal phase contributes 7.17 million CNY/year. This scenario reflects a cost structure heavily focused on upfront construction investment, with moderate operational and disposal expenses. Similarly, in Scenario 2 Guizhou, the construction phase dominates, making up 75.38% of the total lifecycle cost, or 51.44 million CNY/year. The operation phase is significantly lower, comprising 14.62% of the total, or 9.95 million CNY/year, indicating higher cost efficiency in operation compared to Shenzhen. The disposal phase, consistent with the assumption, accounts for 10% of the total, equating to 6.81 million CNY/year. This suggests that, like Shenzhen, Guizhou's lifecycle costs are primarily driven by initial construction, with relatively low operational expenses. Scenario 3 North Virginia, on the other hand, exhibits a more balanced cost distribution between the construction and operation phases. The construction phase accounts for 49.11% of the total lifecycle cost, evaluated at 51.44 million CNY/year, while the operation phase makes up 40.89%, totaling 42.83 million CNY/year. As in the other scenarios, the disposal phase is fixed at 10%, resulting in the highest disposal cost of the three regions, at 10.47 million CNY/year. This distribution indicates that North Virginia experiences much higher operational costs relative to both Shenzhen and Guizhou, reflecting potential differences in energy usage, maintenance needs, or regional operational dynamics.

This analysis highlights significant regional variations in cost structures. While Scenario 1 Shenzhen and Scenario 2 Guizhou are characterized by higher upfront construction costs and lower operational expenses, Scenario 3 North Virginia has a more evenly distributed cost profile, with substantially higher operational costs. As previously mentioned, Scenario 2 in Guizhou benefits from a greener energy mix, lower energy prices, reduced Power Usage Effectiveness (PUE), and more cost-effective labor. These indicate that establishing computational infrastructure, such as data centers, in regions with advantages in energy prices, land costs, and labor markets will significantly reduce overall costs.

The total land area of the data center established by China Mobile in Sanming is approximately 5.35 acres (3566 m²), which includes 1000 racks. The total investment for the Sanming data center is 230 million CNY [24]. Using the quantitative formulas for land and equipment costs in the established model, the estimated total initial investment is 212 million CNY. The primary source of error in this estimate arises from the model's exclusion of labor costs associated with the construction process. During the life cycle cost evaluation of the model, the initial investment is typically amortized based on operational time. However, in this case, to maintain consistency with the cost component of Sanming data center, amortization was not applied; instead, partial estimations were made using the model's formulas. The magnitude of the estimated results aligns with the actual cases, exhibiting an error margin of approximately 8%. Therefore, the output of the model demonstrates a reasonable degree of reliability.

2). Emissions

Active emissions are calculated based on total energy usage and regional energy emission factors. When determining total energy usage, the PUE parameters account for the energy use of support components, including the temperature regulation system. These parameters also cover the passive emissions of building, resulting from construction materials, design, and external climate, thereby eliminating the need to double-count the building's passive emissions.

The embodied emission of computing component could be calculated by the total footprint of Dell PowerEdge R710 2U rack server where 90% footprint appears in use-phase, and the rest of footprint present the embodied emission (636 kgCO_2_ equivalent) through construction and disposal phase [33].

The emission results of 3 scenarios are presented in Table 2.

Scenario 3 exhibits the lowest energy consumption (47.682 TWh/year), as previously noted with its minimal PUE (1.2) in three scenarios. The energy mix in Scenario 3 North Virginia is primarily composed of natural gas, nuclear power, and renewable energy sources, all of which are low-carbon energy resources. Additionally, due to local regulations and market changes, coal usage has significantly declined in this region, leading Virginia's energy structure to shift towards cleaner and lower-carbon energy sources. Consequently, the emission factor associated with its energy consumption is also the lowest, resulting in the smallest active emissions among the three scenarios.

In the two scenarios in China (Scenario 1and 2), although over half of energy supply of Guizhou (Scenario 2) comes from renewable hydropower, approximately one-third still relies on fossil fuels, resulting in an overall grid emission factor of 0.420 kgCO_2_/kWh. In contrast, Scenario 1 Shenzhen has an even lower proportion of clean and renewable energy in its energy structure, yielding the highest grid carbon emission factor of 0.445 kgCO_2_/kWh.

**Fig. 5:**
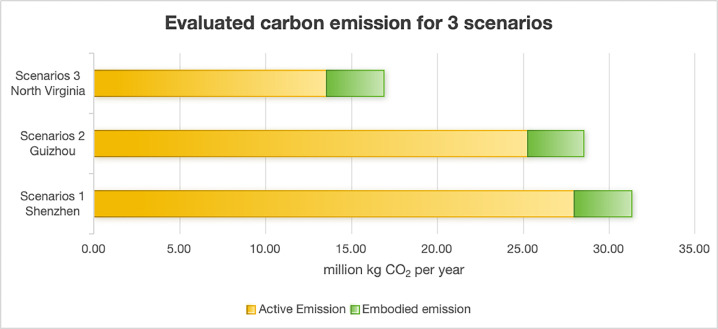
Evaluated carbon emission for 3 scenarios (million kg CO_2_ per year).

**Fig. 6:**
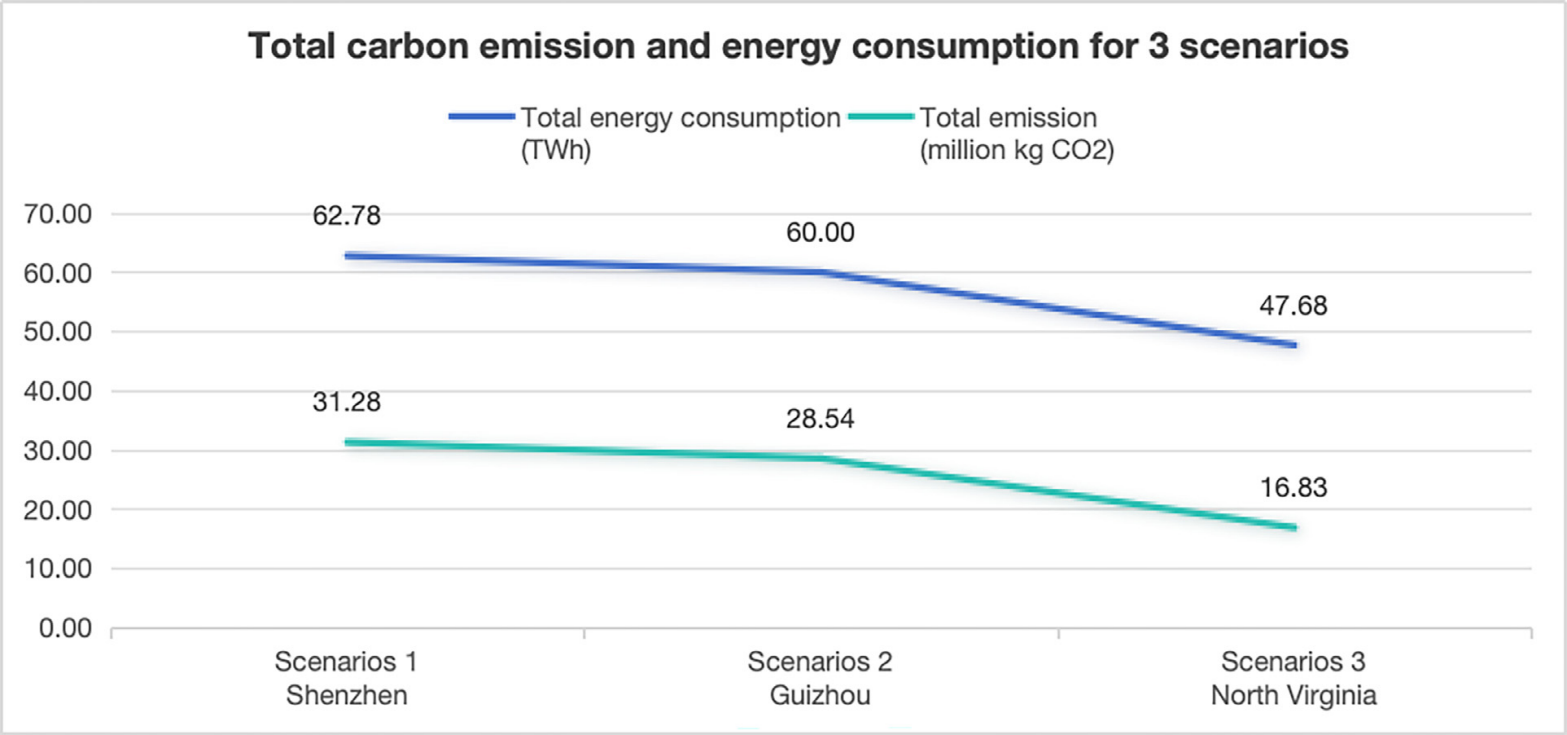
Total carbon emission and energy consumption for 3 scenarios (million kg CO_2_ /TWh per year).

The regional energy structure significantly influences the active carbon emissions produced from electricity consumption. The embodied carbon remains consistent across the three scenarios. According to the data in the table, electricity-related carbon emissions account for approximate 90% of the total carbon emissions in China's Scenarios 1 and 2, while in the U.S. Scenario 3, they account for approximate 80% (Fig. 5). Therefore, carbon emissions generated from energy consumption dominate the overall carbon emissions of the data centers (Fig. 6).

According to the quantitative estimation results (Table 2), the total carbon emission for Scenario 1 Shenzhen amount to 31.277 million kg CO_2_ per year, which is highest in three scenarios, the total carbon emission for Scenario 2 Guizhou is 28.539 million kg CO_2_ per year and there is only 16.833 million kg CO_2_ per year for Scenario 3 North Virginia.

The quantitative model of this study provides a foundational framework and computational cases for the numerical analysis of cost and carbon emissions of computing infrastructure. Concerning lifecycle cost, the model developed in this study decomposes and quantifies the components of cost. In terms of environmental impact, the model considered the active emission of energy usage, passive emission of building and embodied emission for construction and disposal phases.

## Suggestions for application of this method and future studies

The Comprehensive Lifecycle Cost and Environmental Impact Analysis Model developed in this paper quantifies the lifecycle costs and carbon emissions of computing infrastructure. This model covers the three main phases: construction, operation, and disposal. The model thoroughly considers active energy-related carbon emissions, passive emissions from building materials and design, and embodied emissions of components. Compared to traditional approaches that focus solely on the operation phase and energy-related carbon emissions, this proposed model offers a more comprehensive and sustainability-oriented conceptual framework, along with a practical methodology for validation.

Based on the lifecycle cost and carbon emission evaluated results of these scenarios, it can be preliminary concluded that developing digital infrastructure, such as IDC, in regions with low land costs, abundant clean energy, high labor cost-effective—and subsequently redistributing computing capability to areas with higher energy emission factors and higher land costs—can effectively reduce overall carbon emissions and optimize total costs.

The proposed model serves as a quantitative tool for decision-making regarding the allocation and scheduling of computing resources across different regions, as well as for the management and trading of carbon emission quotas. Key components such as land costs and active carbon emissions during the operation phase, which are influenced by regional energy structures, are included in the model's quantification. To address data insufficiencies, the cost and emission evaluations based on this model employ techniques such as equivalent substitution and proportional back-calculation, with these estimations conducted primarily as per benchmark data. Future research could significantly enhance the accuracy of the model through the incorporation of more comprehensive real-time measurement data. With improved cost structure and carbon emission quantifications, the model's results would yield reliable and precise information for decision-making in environmental impact assessments and cost optimization.

## Ethics statements

This work does not involve human subjects, animal experiments, or data collected from social media platforms. Ethical best practices were followed in all cases.

## CRediT authorship contribution statement

**Kezhuo Ma:** Conceptualization, Methodology, Validation, Data curation, Writing – original draft, Writing – review & editing. **Yu Zhou:** Conceptualization, Methodology, Resources, Writing – review & editing, Supervision, Project administration, Funding acquisition.

## Declaration of competing interest

The authors declare that they have no known competing financial interests or personal relationships that could have appeared to influence the work reported in this paper.

## Data Availability

The data supporting the findings of this study are available within the article.

## Appendix Supplementary materials

Image, application 1
