# Supplementary material for: A comprehensive quantitative lifecycle cost and environmental impact analysis model for computing infrastructure
Source: MethodsX. 2024 Oct 24;13:103009. doi: 10.1016/j.mex.2024.103009 (PMC11550342; doi:10.1016/j.mex.2024.103009)
Supplement: Supplementary file 1 [file mmc1.docx]

**Appendix**

**Table 1**. key factors of passive emission

| **Key Factors** | **Description** |
| --- | --- |
| 1. **Building Envelope Characteristics** | |
| Thermal Insulation (R-value or U-value) | The ability of walls, roofs, and floors to resist heat flow. |
| Window-to-Wall Ratio (WWR) | The proportion of window area relative to the total wall area, influencing heat gain and loss. |
| Thermal Mass | The capacity of building materials to absorb and store heat energy. |
| Air Tightness | The capacity of building materials to absorb and store heat energy. |
| 1. **Building Orientation and Geometry** | |
| Solar Exposure | The amount of solar radiation the building receives based on its orientation. |
| Shading Devices | The effectiveness of shading in reducing unwanted solar gain. |
| 1. **Climate Conditions** | |
| External Temperature | The temperature difference between the inside and outside of the building. |
| Solar Radiation | The intensity and angle of solar radiation received by the building. |
| 1. **Material Properties** | |
| Emissivity | The ability of a material to emit absorbed energy. |
| Albedo | The reflectivity of the building surface, which impacts the amount of absorbed solar energy. |

**Table 2.** List of data for computing infrastructure cost and environmental impact quantification.

| **Item** | **Parameter** | **Notes** |
| --- | --- | --- |
| Computing component | Number |  |
|  | Model |  |
|  | Power | Unit W |
|  | Working load | % |
|  | Price | Unit $ |
|  | Computing capability | Unit FLOPS (with FP32) |
| Computing infrastructure | number and size of Computing component | related with land use |
|  | Power Usage Efficiency (PUE) |  |
| Land use | Location |  |
|  | Area of land use | Unit m^2^ |
|  | Price of industrial land use | Unit $/m^2^  (related with location) |
| Electricity | Regional electricity price | Unit $/kwh  (related with location) |
| Emission factor | Regional electricity emission factor | Unit eq CO_2_ kg/kwh  (related with location) |

**Table 3.** Provincial power grid transmission and distribution price (excluding Shenzhen) in 2023[29]

| **No.** | **Region** | **Reactive electricity price**  **CNY/kWh** | | | | | **Capacity electricity price**  **CNY/kW·month** | | | |
| --- | --- | --- | --- | --- | --- | --- | --- | --- | --- | --- |
|  |  | <1kV | 1~10(20)kV | 35kV | 110kV | >220kV | 1~10(20)kV | 35kV | 110kV | 220>kV |
| 1 | Beijing |  | 0.2065 | 0.166 | 0.166 | 0.151 | 51 | 48 | 48 | 45 |
| 2 | Tianjin - general | 0.2839 | 0.251 | 0.1866 | 0.1536 | 0.1316 |  |  |  |  |
|  | Tianjin - industrial | 0.2158 | 0.1687 | 0.1456 | 0.1316 | 0.1102 | 41.6 | 38.4 | 38.4 | 35.2 |
| 3 | Hebei |  | 0.1533 | 0.1333 | 0.1133 | 0.0933 | 35 | 35 | 32 | 32 |
| 4 | Jibei |  | 0.1292 | 0.1132 | 0.0972 | 0.0912 | 37.3 | 37.3 | 34.6 | 34.6 |
| 5 | Shanxi |  | 0.104 | 0.074 | 0.049 | 0.029 | 36 | 36 | 33.6 | 33.6 |
| 6 | the eastern Inner Mongol |  | 0.1483 | 0.1413 | 0.1019 | 0.0789 | 32.8 | 32.8 | 31.2 | 31.2 |
| 7 | the weaster Inner Mongol |  | 0.0795 | 0.0645 | 0.0525 | 0.0425 | 32.8 | 32.8 | 31.2 | 31.2 |
| 8 | Liaoning |  | 0.1024 |  | 0.0838 | 0.0571 | 36.8 |  | 35.2 | 33.6 |
| 9 | Jilin |  | 0.1497 |  | 0.1197 | 0.1097 | 36.8 |  | 35.2 | 35.2 |
| 10 | Heilongjiaing |  | 0.1358 | 0.1144 | 0.1016 | 0.0753 | 36.8 | 36.8 | 35.2 | 35.2 |
| 11 | Shanghai-general | 0.1456 | 0.1272 | 0.0956 | 0.0652 | 0.0551 | 40.8 | 40.8 | 38.4 | 38.4 |
|  | Shanghai-industrial | 0.2234 | 0.2039 | 0.1547 | 0.1251 | 0.1127 | 40.8 | 40.8 | 38.4 | 38.4 |
| 12 | Jiangsu |  | 0.1357 | 0.1107 | 0.0857 | 0.0597 | 51.2 | 48 | 44.8 | 41.6 |
| 13 | Zhejiang |  | 0.126 | 0.0955 | 0.0791 | 0.0688 | 48 | 44.8 | 41.6 | 38.3 |
| 14 | Anhui |  | 0.1428 | 0.1175 | 0.0924 | 0.0673 | 48 | 45.6 | 44 | 40.8 |
| 15 | Fujian |  | 0.1292 | 0.1092 | 0.0842 | 0.0592 | 40 | 39 | 38 | 37 |
| 16 | Jiangxi |  | 0.1505 | 0.1355 | 0.1205 | 0.1105 | 42.3 | 40.6 | 39.1 | 37.5 |
| 17 | Shandong |  | 0.1491 | 0.1341 | 0.1191 | 0.1041 | 38.4 | 35.2 | 35.2 | 32 |
| 18 | Henan |  | 0.168 | 0.1456 | 0.121 | 0.103 | 40 | 36.9 | 33.7 | 30.5 |
| 19 | Hubei |  | 0.1263 | 0.1065 | 0.0884 | 0.0694 | 42 | 42 | 39 | 39 |
| 20 | Hunan |  | 0.1694 | 0.1394 | 0.1104 | 0.0852 | 33.8 | 33.8 | 30.6 | 30.6 |
| 21 | Guangdong |  | 0.0985 | 0.0734 | 0.0734 | 0.0457 | 36.1 | 31 | 31 | 26.1 |
| 22 | Guangxi |  | 0.1476 | 0.1054 | 0.0777 | 0.0288 | 38.7 | 37.3 | 34.2 | 32 |
| 23 | Hainan |  | 0.135 | 0.0815 | 0.0798 | 0.07 | 35.2 | 35.2 | 35.2 | 35.2 |
| 24 | Chongqing |  | 0.1529 | 0.1271 | 0.1078 | 0.0885 | 35.2 | 35.2 | 32 | 32 |
| 25 | Sichuan |  | 0.139 | 0.1092 | 0.0669 | 0.0478 | 35 | 32 | 27 | 24 |
| 26 | Guizhou |  | 0.128 | 0.1143 | 0.0777 | 0.0529 | 35 | 33 | 31 | 30 |
| 27 | Yunnan |  | 0.1296 | 0.1045 | 0.0749 | 0.0555 | 38.4 | 38.4 | 36.8 | 36.8 |
| 28 | Shaanxi (excluding Yulin) |  | 0.1231 | 0.1031 | 0.0831 | 0.0731 | 35.2 | 35.2 | 32 | 32 |
| 29 | Shaanxi (Yulin) |  | 0.1038 | 0.0838 | 0.0638 | 0.0538 | 35.2 | 35.2 | 32 | 32 |
| 30 | Gansu |  | 0.1028 | 0.0888 | 0.0764 | 0.0658 | 38.4 | 36.8 | 32.8 | 32.8 |
| 31 | Qinghai |  | 0.0834 | 0.00779 | 0.0677 | 0.0577 | 33.6 | 33.6 | 32 | 32 |
| 32 | Ningxia |  | 0.092 | 0.0769 | 0.06 | 0.0521 | 28.8 | 28.8 | 25.6 | 25.6 |
| 33 | Xinjiang |  | 0.1204 | 0.11 | 0.0815 | 0.0486 | 32 | 32 | 30.4 | 30.4 |

**Table 4.** Provincial power grid transmission and distribution price (Shenzhen) in 2023 [29]

| Electricity classification | | Reactive electricity price | | | | | Capacity electricity price | |
| --- | --- | --- | --- | --- | --- | --- | --- | --- |
|  |  | 10kV High supply and high meter | 10kV High supply and low meter （380V/220V） | 20kV | 110kV | 220>kV | Maximum capacity (CNY/kW·month) | Transformer capacity （CNY/kVA·month） |
| 1. Industrial and commercial electricity  (101 to 3000 kVA) | Electricity consumption  <250kWh/kA·month | 0.1804 | 0.2054 | 0.1744 | 0.1554 | 0.1304 | 48 | 22 |
|  | Electricity consumption  >250kWh/kA·month | 0.1604 | 0.1854 | 0.1544 | 0.1354 | 0.1104 |  |  |
| 2. Industrial and commercial electricity （>3001kVA） | Electricity consumption  <400kWh/kA·month | 0.1304 | 0.1554 | 0.1254 | 0.1054 | 0.0804 | 42 | 32 |
|  | Electricity consumption  >400kWh/kA·month | 0.1104 | 0.1354 | 0.1044 | 0.0854 | 0.0604 |  |  |
| 3. Industrial and commercial electricity （<100kVA） | |  | 0.2384 |  |  |  |  |  |
